# The Mechanisms, Research Status, and Future Prospects of m6A Modification in Breast Cancer

**DOI:** 10.1002/jgm.70014

**Published:** 2025-02-19

**Authors:** Xiu Xue‐mei, Chen Yang, Ju Wen‐ting, Qin Wen‐xing

**Affiliations:** ^1^ Department of Medical Oncology Fudan University Shanghai Cancer Center Shanghai China; ^2^ Department of Oncology, Shanghai Medical College Fudan University Shanghai China; ^3^ Department of Oncology The Second People's Hospital of Kashi Kashi China; ^4^ Phase I Clinical Trial Center, Department of Medical Oncology, Shanghai Medical College Fudan University Shanghai Cancer Center Shanghai China; ^5^ Department of Medical Oncology Fudan University Shanghai Cancer Center Xiamen Hospital Xiamen Fujian China

**Keywords:** breast cancer, drug resistance, m6A modification, regulatory factors, RNA metabolism

## Abstract

N6‐methyladenosine (m6A) modification is a significant methylation alteration frequently observed in eukaryotic RNAs, garnering considerable attention in the field of breast cancer research in recent years. The m6A modification profoundly influences the onset, progression, and prognosis of breast cancer by regulating RNA stability, translation efficiency, and degradation processes. Numerous studies have demonstrated that m6A regulatory factors, including METTL3, METTL14, and ALKBH5, play pivotal roles in breast cancer cells, affecting cell proliferation, metastasis, and drug resistance. Furthermore, the interactions between m6A modification and non‐coding RNAs, as well as its role in the tumor microenvironment, have increasingly attracted researchers' interest. Although numerous studies have elucidated the dual roles of m6A in breast cancer, its specific molecular mechanisms remain to be thoroughly investigated. Future research should explore various aspects, including the role of m6A in different subtypes of breast cancer, its involvement in chemotherapy resistance, and its interactions with the tumor microenvironment. This exploration will contribute to advancements in the diagnosis and treatment of breast cancer. The present article aims to systematically summarize the research progress on m6A modification in breast cancer, offering novel insights and strategies for future related research and clinical applications.

## Graphical abstract

N6‐methyladenosine (m6A) modification in eukaryotic RNAs has drawn much attention in breast cancer research recently. It impacts breast cancer's onset, progression, and prognosis via regulating RNA‐related processes. m6A regulatory factors play key roles. Also, its interactions with non‐coding RNAs and in the tumor microenvironment are of interest. Despite some known dual roles, its mechanisms need further study. This article summarizes the research to aid future research and clinical applications.

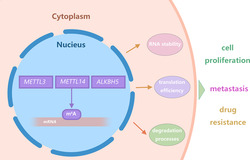

## 1 Background

The N6‐methyladenosine (m6A) modification is a prevalent RNA methylation modification in eukaryotes. In recent years, it has attracted considerable attention for its role in regulating RNA metabolism, gene expression, and cellular functions. The m6A modification not only influences the stability, splicing, transport, and translation of RNA but also plays a critical role in a variety of biological processes, including cell proliferation, differentiation, and apoptosis [1, 2]. In tumor biology, the abnormal expression of m6A modification is closely linked to the occurrence, progression, and prognosis of various tumors.

Breast cancer is the most common malignant tumor in women. Although current treatment methods have been constantly improving, some patients still face challenges in disease control [1]. Research shows that m6A modification plays an important role in the occurrence and development of breast cancer. Its regulatory factors can affect the progression, prognosis, and drug tolerance of breast cancer by influencing RNA metabolism and tumor‐related signaling pathways [3]. In addition, the abnormal expression of m6A modification is associated with the proliferation, transfer, and angiogenesis of breast cancer cells, suggesting that it may become a new therapeutic target for breast cancer [2].

This review aims to systematically explore the role of m6A modification in breast cancer and analyze its regulatory mechanisms and clinical significance. By sorting out relevant literature, we hope to provide new ideas and methods for the early diagnosis and individualized treatment of breast cancer. This research will not only help to deeply understand the biological function of m6A modification in breast cancer but also may provide a theoretical basis for the development of new treatment strategies.

## 2 Main Text

In recent years, N6 methyladenosine (m6A) modification, a pivotal RNA modification, has been garnering growing attention. This modification is accomplished by the addition of a methyl group to the adenosine residue within the RNA molecule. The process is orchestrated by a set of specific enzymes, commonly known as “writers,” “erasers,” and “readers.” Their dynamic interplay endows RNA modification with the capacity to regulate gene expression and modulate multiple cellular biological processes.

The m6A modification exerts a pivotal regulatory function in multiple facets of RNA biology, including RNA stability, splicing, translation, and degradation. Accumulating evidence from studies has demonstrated that m6A modification can govern crucial cellular processes such as cell growth, differentiation, and stress responses by dictating the fate of RNA molecules. Notably, in the context of tumorigenesis and cancer progression, aberrations in m6A modification have been closely linked to the onset and development of a wide array of malignancies. For instance, emerging research has revealed that alterations in m6A modification are potentially associated with the initiation, progression, and metastasis of breast cancer. These findings not only offer a novel theoretical framework but also present innovative strategies for the prognosis assessment and therapeutic intervention of cancer, as elaborated in references [4, 5, 6].

In the realm of breast cancer research, the significance of N6‐methyladenosine (m6A) modification is especially pronounced. m6A modification not only exerts a profound impact on the proliferation and migratory capabilities of tumor cells but also has the potential to propel the development of breast cancer. It does so by modulating immune responses within the tumor microenvironment and orchestrating cell–cell interactions. Additionally, aberrations in m6A modification can potentially drive breast cancer progression by influencing the expression of oncogenes and tumor suppressor genes, positioning it as a promising therapeutic target, as elucidated in references [5, 6]. Moreover, with the deepening of our understanding of m6A modification, the development of specific inhibitors targeting the enzymes involved in m6A‐related processes has been progressing steadily. This advancement holds great promise for the targeted therapy of breast cancer. By precisely regulating the state of m6A modification, there is a realistic possibility of effectively intervening in the trajectory of breast cancer in the future, ultimately leading to improved prognoses for patients, as supported by references [4, 5].

## 3 The Molecular Mechanisms of m6A Modification

The mechanism by which N6‐methyladenosine (m6A) modification exerts its effects in breast cancer cells has increasingly come under the spotlight. As a crucial form of RNA modification, m6A is intricately involved in the regulation of multiple biological processes, such as gene expression, cell proliferation, and migration. In the context of breast cancer, m6A modification manifests its functions via diverse enzymes and proteins.

In recent years, a wealth of studies have unequivocally demonstrated that the methyltransferase METTL3 assumes a pivotal role in the pathogenesis of breast cancer. Elevated expression of METTL3 potentiates the m6A modification of specific mRNAs, thereby propelling the development of breast tumors. Specifically, upon stimulation by epidermal growth factor (EGF), the transcription factor STAT5B engages in an interaction with METTL3. STAT5B then recruits METTL3 to the gene promoter region, thereby promoting both the transcription and m6A modification of CCND1. Owing to the m6A modification, the translation efficiency of CCND1 is enhanced, which in turn bolsters cell proliferation, as detailed in reference [7].

Furthermore, METTL14 exhibits substantial regulatory impacts within breast cancer cells. METTL14 facilitates the expression of USP22 and estrogen receptor α (ERα) by augmenting the mRNA stability of these molecules. This, in turn, exerts a profound influence on the growth and migration of breast cancer cells. The binding of METTL14 to the mRNAs of USP22 and ERα, accompanied by the m6A modification of the latter, further propels the expression of the cell cycle–related protein Cyclin D1. This process gives rise to the METTL14–USP22–ERα axis, which has emerged as a pivotal pathway in the progression of breast cancer, as elucidated in reference [8].

Another pivotal m6A–associated protein is METTL16. The overexpression of METTL16 confers enhanced stability to FBXO5 mRNA. FBXO5 is of great significance in cell proliferation and metastasis. METTL16 bolsters the stability of FBXO5 via m6A modification, thereby propelling the malignant progression of breast cancer, as detailed in reference [9].

In triple‐negative breast cancer (TNBC), FOXM1 has garnered substantial attention owing to its critical role in tumor cell proliferation and metastasis. The expression level of FOXM1 is markedly elevated in TNBC, and its expression is intricately linked to m6A modification. The upregulation of FOXM1 expression by YTHDC1 is contingent upon m6A modification. This discovery underscores that FOXM1 and its associated m6A regulatory network assume a pivotal role in the progression of TNBC, as elaborated in reference [10].

Notably, m6A modification is ubiquitously present in breast cancer and is intimately associated with the expression regulation of non‐coding RNAs (ncRNAs). These ncRNAs not only exert an impact on the biosynthesis and function of m6A but also play a pivotal part in tumor‐related signaling pathways. This implies the significance of the m6A–ncRNA regulatory network in the progression of breast cancer, as elucidated in reference [11].

Meanwhile, ALKBH5, functioning as an m6A demethylase, exerts an influence on the chemotherapy resistance of TNBC cells by modulating the stability of FOXO1 mRNA. Notably, its expression level exhibits a significant correlation with chemotherapy resistance and patient prognosis. This discovery thus proffers a novel potential target for the treatment of breast cancer, as detailed in reference [12].

In summary, the mechanism of m6A modification within breast cancer cells is intricate, entailing the interplay of multiple enzymes and transcription factors. Unraveling these mechanisms not only contributes to uncovering the genesis and development process of breast cancer but also holds the promise of furnishing novel potential therapeutic targets.

## 4 Classification of m6A Regulatory Factors

In breast cancer research, as one of the major RNA modifications, the classification and functions of the regulatory factors of m6A modification are of paramount importance. m6A regulatory factors can be generally categorized into three groups: methyltransferases, demethylases, and binding proteins. These factors are deeply implicated in the initiation and progression of breast cancer, orchestrating a series of biological processes that drive tumorigenesis.

### 4.1 Methyltransferases

Methyltransferases serve as the primary catalytic enzymes for m6A modification, with METTL3 and METTL14 being among the most crucial members. A wealth of studies has demonstrated that METTL3 is upregulated in breast cancer cells. It bolsters the stability of HYOU1 mRNA by augmenting the formation of m6A modification, consequently facilitating the development of multidrug tolerance in these cells. Specifically, METTL3 collaborates with IGF2BP3 to jointly regulate the m6A modification of HYOU1, leading to enhanced resistance to cisplatin treatment, as detailed in reference [13]. This mechanism not only uncovers the pivotal role of METTL3 in breast cancer chemotherapy resistance but also implies that this factor could potentially emerge as a target for breast cancer treatment, offering new insights into therapeutic strategies against this malignancy.

### 4.2 Demethylase

Demethylases, including FTO and ALKBH5, play a pivotal role in modulating the removal of m6A modification. These enzymes exert an influence on the stability and translation efficiency of mRNA by eliminating the m6A modification. Currently, it has been discovered that the downregulation of ALKBH5 results in an elevation of m6A modification in breast cancer cells, thereby fostering the proliferation and migration of tumor cells. Through the regulation of the expression of these demethylases, the biological characteristics of breast cancer cells can be altered, presenting novel treatment strategies, as elaborated in reference [14].

### 4.3 Binding Protein

Binding proteins primarily denote those factors capable of recognizing m6A modification and governing the fate of mRNA, encompassing the YTHDF family and the IGF2BP family. IGF2BP3, an m6A‐binding protein, is upregulated in breast cancer and is intimately associated with tumor malignancy and prognosis. It promotes tumor cell proliferation and drug resistance by enhancing the stability of specific mRNAs, such as HYOU1, as reported in references [13, 15]. Furthermore, the functions of YTHDF proteins have been demonstrated to exert regulatory impacts in breast cancer, influencing mRNA translation and degradation. Notably, RNA modifications may have far‐reaching effects in regulating tumor cell behavior. This not only deepens our understanding of the molecular mechanisms underlying breast cancer but also furnishes novel ideas and strategies for clinical treatment, as elucidated in reference [16].

Consequently, diverse types of m6A regulatory factors assume pivotal roles in the initiation, progression, and modulation of the chemotherapy response in breast cancer. Unraveling the specific mechanisms of these factors not only enables a profound exploration of the molecular biology underpinning breast cancer but also holds the potential to offer novel targets and strategies for clinical intervention. This knowledge could be instrumental in advancing the diagnosis, treatment, and prognosis prediction of breast cancer, thereby improving patient outcomes in the battle against this complex malignancy.

## 5 The Role of m6A Modification in the Onset and Progression of Breast Cancer

m6A, the most prevalent form of RNA methylation modification, has its dysregulation intricately linked to the onset and progression of a diverse range of diseases, with a particularly strong association in breast cancer. A substantial body of research has unequivocally demonstrated that m6A modification exerts a profound influence on the initiation, development, prognosis, and treatment landscape of breast cancer. Firstly, within breast cancer cells, m6A plays a pivotal role in the tumorigenesis and development processes. It does so by modulating RNA metabolism and tumor‐related signaling pathways and is also closely intertwined with tumor drug resistance, as detailed in reference [1]. Simultaneously, the aberrant expression of m6A modification and its associated regulatory proteins can either activate or suppress specific signaling cascades or oncogenes. This, in turn, has a direct impact on the proliferation and metastatic potential of breast cancer cells, as elucidated in reference [3]. Furthermore, m6A modification holds significant potential application value in the prognosis assessment and treatment of breast cancer. It can either promote or impede tumorigenesis, metastasis, and angiogenesis, thereby positioning m6A as a promising potential therapeutic target for breast cancer, as described in reference [2]. Additionally, the evolving detection strategies for m6A‐modified RNA and the emerging understanding of its role in biological processes offer novel perspectives and avenues for future breast cancer treatments, as presented in reference [17].

Secondly, when it comes to breast cancer metastasis, METTL14, functioning as an m6A methyltransferase, has been convincingly demonstrated to promote the growth and migration of breast cancer cells. It achieves this by regulating the stability of ubiquitin‐specific peptidase 22 (USP22) and ERα. A positive correlation exists between the expression levels of METTL14 and those of USP22 and ERα. Notably, the inhibition of METTL14 can markedly suppress the proliferation and migration capabilities of breast cancer cells, as detailed in reference [8]. Moreover, METTL16 is also implicated in the growth and metastasis of breast cancer. It propels the malignant phenotype of breast cancer cells by enhancing the stability of FBXO5 mRNA, as reported in reference [9].

In the context of drug resistance, m6A modification also assumes a pivotal role. METTL3 and IGF2BP3 augment the resistance of breast cancer cells to doxorubicin (Dox) through the regulation of m6A modification of HYOU1. Specifically, within Dox‐resistant breast cancer cells, the expression level of METTL3 is elevated. Intriguingly, the downregulation of METTL3 can reverse this drug‐resistant phenotype, which clearly indicates that METTL3 exerts a promoting effect on Dox resistance, as detailed in reference [13]. Furthermore, the ALKBH5‐mediated demethylation of FOXO1 mRNA plays a critical part in maintaining cancer stem cell characteristics and Dox resistance. This finding highlights the intricate relationship between m6A modification and drug resistance, as elaborated in reference [12]. This complex interplay between m6A‐related factors and drug resistance not only deepens our understanding of the molecular mechanisms underlying chemoresistance in breast cancer but also offers potential new targets for overcoming this challenging aspect of cancer treatment.

Notably, recent studies have revealed significant interactions between m6A modification and ncRNAs. As one of the most prevalent RNA modifications, m6A is ubiquitously present in ncRNAs, exerting a profound influence on their biosynthesis and functions. Moreover, m6A serves as a key regulator in tumor‐related signaling pathways. Concurrently, ncRNAs can also modulate or target m6A modification, thereby playing a pivotal role in cancer progression, as elaborated in reference [11]. Despite the fact that the role of the m6A–ncRNA regulatory mechanism in breast cancer remains incompletely understood, this line of research offers novel perspectives for exploring potential m6A‐modification–based targets in future breast cancer treatments. The intricate relationship between m6A and ncRNAs holds great promise for uncovering new therapeutic strategies, which could potentially revolutionize the management of breast cancer by targeting this previously under‐explored regulatory axis.

In summary, m6A modification is intricately involved in the mechanisms underlying breast cancer metastasis and drug resistance through the regulation of the expression and stability of key genes. Moving forward, future investigations will delve deeper into exploring the potential targets associated with m6A modification in breast cancer treatment. These endeavors are expected to yield novel insights and strategies, thereby offering fresh impetus for the clinical management of breast cancer. This research trajectory not only has the potential to enhance our understanding of the molecular basis of breast cancer but also holds the promise of translating fundamental discoveries into tangible clinical benefits for patients suffering from this complex malignancy.

## 6 Analysis of Consistency and Difference

In the body of literature addressing the impact of m6A modification on breast cancer, while a number of studies indicate that m6A modification promotes the initiation and progression of breast cancer, there are also divergent viewpoints and findings. These consistencies and discrepancies merit in‐depth analysis. Such an analysis could potentially uncover underlying molecular mechanisms, clarify the complex role of m6A modification in breast cancer biology, and offer more accurate insights for future research directions and therapeutic strategies.

The upregulated expression of METTL3 potentiates the m6A modification of specific mRNAs, thereby assuming a pivotal role in breast tumorigenesis. METTL3 engages in an interaction with the transcription factor STAT5B and recruits itself to gene promoters, such as that of CCND1. This process consequently exerts a profound influence on cell proliferation, as detailed in reference [7]. This mechanism reveals the importance of m6A modification in regulating the growth of breast cancer cells. Moreover, in high‐grade breast tumors, the expression of p‐STAT5B is positively correlated with that of METTL3. Conversely, METTL14 augments the mRNA expression of USP22 and ERα via m6A modification, thereby facilitating the growth and migration of ERα+ breast cancer cells, as reported in reference [8]. These distinct regulatory mechanisms suggest that m6A modification may elicit differential effects on diverse targets. This, in turn, contributes to the heterogeneity and diversity characteristic of breast cancer, highlighting the complexity of the molecular mechanisms underlying this malignancy.

Furthermore, m6A modification is intimately associated with the initiation, progression, and drug resistance of breast cancer. However, the mechanism by which m6A regulatory factors exert their influence on breast cancer remains incompletely understood, as indicated in reference [1]. This perspective serves as a complementary addition to the previous two studies, underscoring the significance of m6A modification in breast cancer biology. Nevertheless, it falls short of clearly delineating the specific regulatory pathways involved. Unraveling these pathways is crucial for a more comprehensive understanding of the role of m6A in breast cancer and may hold the key to developing novel therapeutic strategies targeting this modification.

Regarding drug effects, research findings have demonstrated that cisplatin treatment elevates the levels of m6A modification factors, whereas TNF‐α treatment leads to a reduction in the expression of these factors. This clearly indicates that distinct types of treatments can impact the levels of m6A modification factors. It implies that the regulatory mechanism of m6A modification is likely influenced by multiple factors, including cell types and external stimuli, as detailed in reference [16]. This observed difference further uncovers that, within the realm of breast cancer research, the biological functions of m6A modification are probably complex and multifaceted, as elaborated in reference [18]. Understanding these nuances is crucial for comprehensively grasping the role of m6A modification in breast cancer, which may in turn offer novel insights into therapeutic strategies that can potentially manipulate this modification to combat the disease.

While existing studies have achieved a certain level of consensus on some of the mechanisms underlying the impact of m6A modification on breast cancer, disparities persist in specific regulatory processes and biological functions. These divergences can potentially be attributed to factors such as variations in experimental designs, the utilization of different cell models, and the diverse mRNA targets under investigation. Consequently, further in‐depth exploration of m6A modification is imperative to comprehensively elucidate its mode of action in breast cancer. Such investigations could involve more standardized experimental protocols, a broader range of cell models representative of different breast cancer subtypes, and a more systematic analysis of mRNA targets. By doing so, we can gain a more complete understanding of how m6A modification contributes to breast cancer development, progression, and response to treatment, ultimately paving the way for the development of more effective therapeutic strategies.

## 7 Research Trends and Gap Analysis

### 7.1 Current Research Trends

In recent years, alterations in the functions and expressions within the realm of epigenetic regulation have emerged as a focal point in cancer research. Notably, during the development and metastasis of breast cancer, changes in epigenetic modification are considered a driving factor, as detailed in reference [4]. Furthermore, the aberrant expression of m6A and the dysregulation of associated regulatory proteins have the capacity to either activate or suppress specific signaling pathways or oncogenes. This, in turn, exerts a profound influence on the proliferation, metastasis, and prognosis of breast cancer, as elucidated in reference [3]. Understanding these epigenetic intricacies is crucial for unraveling the complex molecular mechanisms underlying breast cancer progression. It may also offer novel therapeutic targets and strategies for the management of this prevalent malignancy, potentially leading to improved patient outcomes in the future.

### 7.2 Existing Research Gaps

Despite a substantial body of research having delved into the role of m6A modification in breast cancer, several critical research lacunae persist. Firstly, the molecular mechanisms through which m6A RNA methylation governs the initiation and progression of breast cancer remain incompletely understood. Although the functions of m6A regulatory factors have been collated, the precise regulatory mechanisms necessitate further in‐depth exploration. This knowledge gap restricts our holistic comprehension of their roles in breast cancer, as highlighted in reference [3]. Simultaneously, m6A functions as a “double‐edged sword” in breast cancer, with the capacity to both promote and inhibit tumorigenesis. Nevertheless, investigations into the specific mechanisms underlying these dual roles are still scanty, as pointed out in reference [2]. Current research predominantly centers on the individual effects of m6A regulatory factors, lacking a systematic analysis of their interactions and network‐level impacts. This shortcoming poses a formidable challenge to the formulation of clinical treatment strategies. Addressing these research gaps is crucial for advancing our understanding of breast cancer biology and developing more effective, targeted therapies.

## 8 Limitations of the Study

### 8.1 Methodological Limitations

In the contemporary research exploring the relationship between m6A modification and breast cancer, methodological constraints have markedly influenced the reliability and reproducibility of research outcomes. Primarily, a substantial number of studies predominantly hinge on cell lines and animal models. These experimental systems, while valuable, may not comprehensively mirror the intricate nature of human tumors. For instance, in 2024, Shaocheng Zhou et al. elucidated the role of METTL3/IGF2BP3‐regulated m6A modification in the resistance of breast cancer cells to Dox via cell‐based experiments. Nevertheless, it is crucial to note that these findings cannot be directly translated to patients with diverse subtypes of breast cancer in clinical scenarios, as reported in reference [13]. This discrepancy between experimental models and real‐world clinical situations underscores the need for more refined research methodologies that can bridge this gap, enabling a more accurate understanding of the role of m6A modification in human breast cancer and facilitating the development of more effective therapeutic strategies.

Secondly, sample size and selection bias represent critical limiting factors within the realm of research on the relationship between RNA modifications, particularly m6A, and breast cancer. In the majority of investigations, the sample sizes employed tend to be relatively small, and the process of sample selection may lack sufficient representativeness. The relationship between RNA modification and tumors is inherently intricate and dynamic. An imbalance in the samples can introduce significant biases into research conclusions, especially when attempting to establish correlations between m6A modification and clinical features, as highlighted in reference [14]. This issue not only undermines the reliability of the findings but also restricts the generalizability of the research outcomes across diverse patient populations. To address this, future studies should prioritize the implementation of more rigorous sampling strategies, ensuring larger, more diverse, and representative sample sets. This approach is essential for enhancing the validity and robustness of research on the role of m6A in breast cancer, enabling more accurate insights into its clinical implications and potentially leading to more effective therapeutic strategies.

Moreover, the constraints of technical methodologies wield a significant impact on research outputs. Currently, although RNA sequencing and other high‐throughput techniques have become the cornerstone methods for probing m6A modification, they are still marred by limitations in sensitivity and specificity. As elaborated in reference [17], Huiping Sun et al. explored a variety of detection strategies; nonetheless, they failed to completely surmount the problems of errors and non‐reproducibility in the detection of m6A modification. This state of affairs presents a formidable challenge to the comparability of research results across different laboratories and experimental settings. To surmount these obstacles, unremitting efforts are essential for the development of more precise and dependable detection methods. Refinement of existing high‐throughput technologies or the exploration of novel approaches has the potential to augment the accuracy of m6A modification analysis. Such progressions would not only enhance the consistency of research outcomes but also afford a more profound understanding of the role of m6A modification in breast cancer. This, in turn, would expedite the translation of fundamental research findings into practical clinical applications, potentially revolutionizing the diagnosis and treatment paradigms for breast cancer patients.

Finally, a conspicuous deficiency prevalent in numerous studies lies in the inability to comprehensively consider the dynamic alterations of m6A modification and its elaborate interactions with other biological processes. In 2024, Xuan Liu et al. expounded, as detailed in reference [11], that m6A modification not only influences the functions of ncRNAs but also likely acts in unison with other RNA modifications. Regrettably, the research progress in this domain remains distressingly limited. This methodological oversimplification inevitably results in a superficial and inadequate understanding of the multifarious role of m6A in breast cancer development. To achieve a more profound and accurate understanding, future research endeavors must embrace a more holistic perspective. This entails meticulously tracking the dynamic fluctuations of m6A modification across diverse stages of breast cancer progression and systematically exploring its synergistic or antagonistic associations with other biological mechanisms. By undertaking such comprehensive investigations, we can effectively bridge the existing knowledge chasms and lay a solid foundation for the formulation of more targeted and efficacious therapeutic strategies against breast cancer. This approach holds the promise of not only enhancing our fundamental understanding of the disease but also translating this knowledge into tangible clinical benefits, ultimately improving the prognosis and quality of life for patients afflicted with breast cancer.

### 8.2 Data Source Limitations

In the realm of studies delving into the relationship between m6A modification and breast cancer, the limitations of data sources exert a substantial influence on the reliability and generalizability of research conclusions. Certain researchers have conducted evaluations of the expression alterations of m6A modification regulatory factors across diverse cancer cell types, prominently including breast cancer, cervical cancer, lung cancer, and colon cancer, as elucidated in reference [18]. Nevertheless, such cross‐cancer‐type comparisons run the risk of obscuring the distinct and unique mechanisms characteristic of specific cancer types. Each cancer type is endowed with its own molecular landscape, genetic makeup, and microenvironmental context, which can lead to highly specific manifestations of m6A‐related regulatory processes. By lumping together data from multiple cancer types, the nuanced and cancer‐specific features of m6A modification in breast cancer may be overlooked, thereby hampering a comprehensive and in‐depth understanding of the role of m6A in breast cancer biology. This calls for a more focused and cancer‐type‐specific approach to data collection and analysis, enabling a more accurate dissection of the m6A‐related mechanisms underlying breast cancer development, progression, and potential therapeutic responses.

Firstly, the scale and diversity of datasets represent a pivotal concern. In some investigations, reliance on small‐scale samples often ensues, yielding results with insufficient statistical significance. This, in turn, undermines the generalizability of the conclusions drawn, as highlighted in reference [16]. Large‐scale sample data has the potential to offer more robust statistical support. However, currently, a substantial number of extant studies remain concentrated on restricted cell lines and samples. This narrow focus runs the risk of fostering misunderstandings or underestimations of the role that m6A modification plays in breast cancer. Each cell line and sample subset has its own unique characteristics, and a limited dataset may not comprehensively capture the full spectrum of m6A‐related phenomena in breast cancer. To achieve a more accurate and comprehensive understanding, future research should prioritize the acquisition and analysis of large‐scale, diverse datasets. This approach will enable a more precise evaluation of the significance of m6A modification in breast cancer, facilitating the development of more reliable and generalizable insights into the disease's molecular mechanisms and potential therapeutic targets.

Secondly, the lack of consistency in results across different experimental conditions presents a formidable challenge to the accurate interpretation of data. The impacts of employing different inducers, such as cisplatin and TNF‐α, on the expression of m6A modification factors can vary significantly depending on the cell lines utilized. As detailed in reference [18], these diverse outcomes are not necessarily applicable across all cancer types. Given this variability, conclusions derived from a single experimental condition are unlikely to comprehensively mirror the role of m6A modification in the progression of breast cancer. Each cell line has its own distinct genetic background, signaling pathways, and epigenetic landscapes, which can lead to differential responses to the same inducers. This complexity highlights the need for a more systematic and comprehensive approach to experimental design. By conducting experiments across multiple cell lines, cancer types, and a range of experimental conditions, researchers can better capture the true nature of m6A modification's involvement in breast cancer progression. Such an approach will enhance the reliability and generalizability of research findings, ultimately contributing to a more profound understanding of the underlying molecular mechanisms and potentially enabling the development of more targeted therapeutic strategies.

Moreover, the method of data acquisition is an aspect that demands close attention. For instance, while the utilization of high‐throughput sequencing technology has the potential to furnish copious information regarding RNA modification, the interpretation of the resulting data is heavily reliant on intricate bioinformatics analysis. This necessitates that researchers be equipped with the corresponding analytical capabilities and tools. Nevertheless, as indicated in reference [19], this requisite expertise and infrastructure may be lacking in certain studies. Inadequate data processing and analysis can give rise to misinterpretations of the biological functions of m6A modification. Such misunderstandings can then cascade to undermine the reliability of research findings. The complexity of high‐throughput sequencing data, with its vast amount of raw information, requires meticulous handling. Incorrect analysis may lead to spurious associations being drawn between m6A modification and various biological processes in breast cancer or may cause the overlooking of true relationships. To ensure the integrity of research on m6A modification in breast cancer, it is imperative that researchers invest in developing or accessing appropriate bioinformatics resources and training. This will enable them to accurately distill meaningful biological insights from the high‐dimensional data, thereby enhancing the validity and impact of their research.

## 9 Future Research Directions

### 9.1 The Relationship Between m6A Modification and Breast Cancer

In future research endeavors exploring the relationship between m6A modification and breast cancer, a novel and highly worthy direction of focus lies in conducting in‐depth explorations into the mechanism of action of m6A modification within distinct breast cancer subtypes. Paramount among these investigations is elucidating how m6A modification governs the biological characteristics of breast cancer cells. Existing research has demonstrated that m6A modification exerts a dual‐function role in the initiation, progression, and metastasis of breast cancer, as detailed in reference [2]. Given this, a crucial question emerges: Can systematic genomics and transcriptomics studies clarify the specific roles of m6A modification in different subtypes, such as TNBC and hormone receptor‐positive breast cancer? Unraveling how m6A regulatory factors interact with other molecules in these subtypes is essential. This line of inquiry holds the potential to help us identify potential biomarkers and therapeutic targets. By comprehensively mapping the m6A‐related molecular networks in diverse breast cancer subtypes, we can gain a more nuanced understanding of the disease's heterogeneity at the molecular level. This, in turn, may lead to the development of more personalized and effective treatment strategies tailored to the unique biology of each breast cancer subtype, thereby improving patient outcomes and revolutionizing the landscape of breast cancer management.

Furthermore, delving deep into the mechanism through which m6A modification influences chemotherapy resistance represents an equally crucial research direction. Prior investigations, as detailed in reference [12], have indicated that the m6A demethylase ALKBH5 is implicated in the chemotherapy resistance of TNBC by modulating the stability of FOXO1 mRNA. In future research, efforts could be concentrated on how to precisely target the molecular pathways associated with m6A modification. Initiating from the development of multiple models related to m6A modification, a comprehensive evaluation of their potential in breast cancer prognosis prediction and treatment can be carried out. By doing so, it becomes possible to surmount chemotherapy resistance, a major hurdle in current breast cancer treatment. This approach holds the promise of enhancing treatment efficacy, improving patient survival rates, and ultimately revolutionizing the clinical management of breast cancer. Such research not only fills the existing knowledge gaps in the field but also paves the way for the development of novel, targeted therapeutic strategies, bringing new hope to breast cancer patients who are currently facing the challenges of chemotherapy resistance.

Finally, investigating the interaction between m6A modification and the tumor microenvironment stands as a research avenue that merits in‐depth exploration. The tumor microenvironment is pivotal in the initiation, progression, and metastasis of tumors. Notably, m6A modification may exert an influence on the biological behaviors of breast cancer by modulating the interactions between tumor cells and immune cells, as well as stromal cells within the microenvironment, as elucidated in reference [4]. Consequently, studying the role of m6A modification in the tumor microenvironment, integrating clinical data with basic research, and probing the application value of m6A as a biomarker hold great promise. This line of research is anticipated to offer novel ideas and methods for the early diagnosis and personalized treatment of breast cancer. By comprehensively understanding how m6A modification impacts the complex ecosystem of the tumor microenvironment, we can identify new diagnostic markers and therapeutic targets. This could lead to more accurate and timely detection of breast cancer at its early stages and enable the development of tailored treatment strategies that take into account the unique molecular characteristics of each patient's tumor microenvironment. Such advancements have the potential to significantly improve the prognosis and quality of life for breast cancer patients and to drive the evolution of breast cancer management towards a more precision‐medicine–based approach.

## 10 Conclusion

In recent research endeavors, m6A modification has emerged as being intricately linked to the initiation, progression, and prognosis of breast cancer. As a prevalent methylation modification in eukaryotic RNA, m6A exerts regulatory effects on multiple facets of RNA metabolism. This encompasses the modulation of RNA stability, translation efficiency, and the processes of RNA degradation. The aberrant expression of m6A modification has been found to be associated with diverse biological processes in breast cancer. These associations strongly suggest that m6A modification may hold substantial clinical significance in the diagnosis, prognosis prediction, and treatment strategies for breast cancer. Unraveling the precise mechanisms by which m6A modification impacts breast cancer biology could potentially open up new avenues for the development of innovative diagnostic tools and targeted therapeutic interventions, thereby improving the clinical management and outcomes for patients with breast cancer.

m6A regulatory factors assume a pivotal role in the progression of breast cancer. The overexpression of METTL16 exhibits a positive correlation with the growth and metastasis of breast cancer cells. It propels the malignant behaviors of breast cancer cells by augmenting the stability of FBXO5 mRNA. Furthermore, the abnormal expression of m6A modification has the capacity to either activate or suppress specific signaling pathways. This, in turn, exerts a profound influence on the proliferation, metastatic spread, and prognosis of breast cancer. These research findings underscore the significance of m6A modification in breast cancer research. They not only deepen our understanding of the molecular mechanisms underlying breast cancer progression but also offer a novel perspective for exploring m6A modification as a potential therapeutic target. Harnessing the knowledge of m6A‐related regulatory mechanisms may pave the way for the development of innovative and more effective treatment strategies tailored to combat breast cancer.

Despite the revelation of the dual‐role nature of m6A in breast cancer by previous studies, the specific molecular mechanisms underpinning these functions remain incompletely understood. A more profound exploration of m6A's actions is thus imperative. Such further research endeavors will be instrumental in attaining a comprehensive comprehension of the role of m6A regulation in the initiation and progression of breast cancer. By delving deeper into these molecular intricacies, we stand to gain novel insights that could potentially translate into innovative strategies and methods for the prevention and treatment of breast cancer. Unraveling the full spectrum of m6A‐related regulatory pathways may open up new avenues for therapeutic interventions, targeting the underlying molecular drivers of breast cancer, and ultimately leading to improved clinical outcomes for patients afflicted with this disease.

## Author Contributions

Writing – original draft: Xiu Xue‐mei. Writing – review and editing: Xiu Xue‐mei. Supervision: Chen Yang and Ju Wen‐ting. Supervision: Qin Wen‐xing. The authors read and approved the final manuscript.

## Ethics Statement

The authors have nothing to report.

## Conflicts of Interest

The authors declare no conflicts of interest.

## Data Availability

Data sharing is not applicable to this article as no new data were created or analyzed in this study.
